# Genome-wide alignment-free phylogenetic distance estimation under a no strand-bias model

**DOI:** 10.1093/bioadv/vbac055

**Published:** 2022-08-12

**Authors:** Metin Balaban, Nishat Anjum Bristy, Ahnaf Faisal, Md Shamsuzzoha Bayzid, Siavash Mirarab

**Affiliations:** Bioinformatics and System Biology Program, University of California San Diego, San Diego, CA 92093, USA; Computer Science and Engineering, Bangladesh University of Engineering and Technology, Dhaka 1205, Bangladesh; Computer Science and Engineering, Bangladesh University of Engineering and Technology, Dhaka 1205, Bangladesh; Computer Science and Engineering, Bangladesh University of Engineering and Technology, Dhaka 1205, Bangladesh; Bioinformatics and System Biology Program, University of California San Diego, San Diego, CA 92093, USA; Electrical and Computer Engineering, University of California San Diego, San Diego, CA 92093, USA

## Abstract

**Summary:** While alignment has been the dominant approach for determining homology prior to phylogenetic inference, alignment-free methods can simplify the analysis, especially when analyzing genome-wide data. Furthermore, alignment-free methods present the only option for emerging forms of data, such as genome skims, which do not permit assembly. Despite the appeal, alignment-free methods have not been competitive with alignment-based methods in terms of accuracy. One limitation of alignment-free methods is their reliance on simplified models of sequence evolution such as Jukes–Cantor. If we can estimate frequencies of base substitutions in an alignment-free setting, we can compute pairwise distances under more complex models. However, since the strand of DNA sequences is unknown for many forms of genome-wide data, which arguably present the best use case for alignment-free methods, the most complex models that one can use are the so-called no strand-bias models. We show how to calculate distances under a four-parameter no strand-bias model called TK4 without relying on alignments or assemblies. The main idea is to replace letters in the input sequences and recompute Jaccard indices between k-mer sets. However, on larger genomes, we also need to compute the number of k-mer mismatches after replacement due to random chance as opposed to homology. We show in simulation that alignment-free distances can be highly accurate when genomes evolve under the assumed models and study the accuracy on assembled and unassembled biological data.

**Availability and implementation:**

Our software is available open source at https://github.com/nishatbristy007/NSB.

**Supplementary information:**

Supplementary data are available at *Bioinformatics Advances* online.

## 1 Introduction

The dominant methodology used in phylogenetic inference is assembling and aligning sequences and using the alignments as input to phylogenetic inference. However, a large body of work also exists on alignment-free (Bogusz and Whelan, 2016; Daskalakis and Roch, 2013; Haubold, 2014; Höhl and Ragan, 2007; Jun *et al.*, 2010; Leimeister *et al.*, 1 2017; Wu *et al.*, 2009) and even assembly-free methods for inferring phylogenies (Allman *et al.*, 2017; Fan *et al.*, 2015; Linard *et al.*, 2019; Sarmashghi *et al.*, 2019; Yi and Jin, 2013). While, for the most part, the alignment-free methods have not been as accurate as alignment-based methods (Bogusz and Whelan, 2016; Höhl and Ragan, 2007), they do provide several benefits and enjoy emerging applications. The most obvious advantage is that inferring alignments is difficult, and forgoing them would simplify the tree inference. The challenges are further exacerbated when working with genome-wide data, where long sequences and large-scale events such as rearrangements further challenge alignment (Zielezinski *et al.*, 2017). There is, therefore, a hope that by skipping the alignment step, we can eliminate the errors (Zielezinski *et al.*, 2017) that can occur in the alignment step and impact phylogenetic accuracy (Wang *et al.*, 2011; Lunter *et al.*, 2008; Ogden and Rosenberg, 2006). In particular, at the whole-genome level, homology detection and alignment are both challenging and error-prone (Earl *et al.*, 2014; Letsch and Kjer, 2011; Springer and Gatesy, 2018). Therefore, it seems possible (though by no means certain) that alignment-free methods could provide a better trade-off between accuracy, running time and complexity of analyses, especially for analyzing genomes (Forsdyke, 2019).

The main advantage of alignment-free methods may come from situations where alignment is not possible. In particular, genome skimming has recently emerged as a promising method of acquiring genome-wide data inexpensively (Bohmann *et al.*, 2020) by generating short reads from across the genome at low coverage (e.g. 1X). While such data cannot be assembled, mapping them against a reference genome, when available (Westbury *et al.*, 2021), or analyzing them in an assembly-free fashion, when references are unavailable, are now possible (Balaban and Mirarab, 2020; Balaban *et al.*, 2020; Lau *et al.*, 2019; Sarmashghi *et al.*, 2019; Tang *et al.*, 2019). Multiple sequence alignment is not possible given the low coverage, leaving us with alignment-free methods as the only option. Many assembly-free methods use *k*-mers to compute distances between all pairs of species and use distance-based methods to infer a phylogeny. A long history (Reinert *et al.*, 2009; Ren *et al.*, 2018; Yi and Jin, 2013) of methods using *k*-mer counts (with small *k*) exists. Some recent *k*-mer-based methods that work with both assembled and unassembled data and model low coverage instead use presence/absence with large *k* (Fan *et al.*, 2015; Sarmashghi *et al.*, 2019; Tang *et al.*, 2019); refer to a recent benchmarking analysis for a complete survey (Zielezinski *et al.*, 2019).

Despite their practical benefits, alignment-free methods have limitations of their own, notably, the reduced complexity of the sequence evolution models employed. Most alignment-free methods rely on the simplest model of sequence evolution, Jukes–Cantor (JC; Jukes and Cantor, 1969), which assumes equiprobable bases and base substitutions. Criscuolo (2019) recently showed how to compute alignment-free distances under the slightly more complex F81 (Felsenstein, 1981) model where the base frequencies can be different. By contrast, alignment-based methods use more complex models, such as the general time-reversible (GTR; Tavaré, 1986) model paired with models of rate variation across sites and further partitioning data to allow changing model parameters. The reliance on models like JC and F81 is not an oversight by the research community. In the absence of alignments, it is more challenging to design methods for more complex sequence evolution models that need to estimate parameters related to relative rates of substitutions among bases. The difficulties are exacerbated by the fact that sequences can come from either of the two strands for unassembled and unaligned data, making it difficult to calculate some parameters of complex models and impossible to compute others (Zagordi and Lobry, 2005). Nevertheless, Sarmashghi *et al.* (2019) proposed a trick that they conjectured could be used in conjunction with the well-known LogDet technique (Steel, 1994) to compute distances under the GTR model from unassembled reads. The claim that distances under more complex time-reversible models like GTR can be computed from unassembled data has never been carefully examined.

Here, we observe that for unassembled input data, where reads can be of either strand, no strand-bias models are the most complex time-reversible models one can employ. We go on to describe an algorithm that can estimate all the parameters needed to compute distances for a time-reversible no strand-bias model called TK4 (Takahata and Kimura, 1981). Our algorithm replaces the nucleotide characters in input sequences in four ways (e.g. C→G) and computes the Jaccard index between these letter-substituted sequences. We then observed that a fundamental assumption of many *k*-mer-based methods (that matching *k*-mers can only appear by homology for a large enough *k*) is often violated after letter substitutions, especially for genomes with unbalanced base frequencies, because the number of characters in the base genomes decreases from four to three. Luckily, the expected number of random matches between two *k*-mers from two random genomes can be derived (Röhling *et al.*, 2020); we go one step further and compute the expected (containment) Jaccard between two unrelated genomes (Lemma 1). Using these calculations, we can correct for the effect of non-homologous *k*-mer matches. We go on to show that using this technique to compute distances under the TK4 model can improve accuracy compared to JC, especially when the distances are high and deviations from the JC model are sufficiently high. We then use biological data to demonstrate that using the TK4 model improves the concordance of phylogenetic trees inferred using alignment-free methods and alignment-based methods, indicating improved accuracy. We end by discussing the limitations of the method.

## 2 Approach

### 2.1 Background information

#### 2.1.1 Evolutionary model

Suppose that we have two homologous DNA sequences G and H on character alphabet Σ={A,C,G,T} taken from two species $F_{1}$ and $F_{2}$ that share a common ancestor. For a given base i∈Σ, let $\bar{i}$ denote its complementary base (e.g. $\bar{A}=T$). We assume that each homologous site in G or H is evolved independently and according to a stationary continuous-time Markov-chain process on state set Σ that is defined by a 4 × 4 instantaneous rate matrix $R=(r_{ij})$. Letting $\pi =\left[\begin{array}{cccc} \pi_{A} & \pi_{C} & \pi_{G} & \pi_{T} \end{array}\right]$ denote the stationary base frequencies in G and H (thus, πR=0), the most general time-reversible stationary model, GTR (Tavaré, 1986), adds local balance constraints (i.e. $\forall i,j:\pi_{i}r_{ij}=\pi_{j}r_{ji}$), which lead to nine free parameters. Another constraint is added by requiring the time to be in the unit of one expected mutation, leaving us with eight free parameters. The transition matrix $P=e^{Rt}$ governs probabilities of base substitutions after time *t*.

We aim to estimate the time of divergence *t* between the two given genomes. Such estimates, if statistically unbiased, would converge to additivity and can be used with any distance-based phylogenetic inference method. In the last 50 years, numerous models with reduced complexity (i.e. fewer parameters) compared to the general Markov model have been proposed (Hasegawa *et al.*, 1985; Jukes and Cantor, 1969; Steel, 1994; Tamura and Nei, 1993), and some of these models have analytical equations for distance calculations (Hasegawa *et al.*, 1985; Tamura and Nei, 1993). For example, let *genomic distance d* be the probability of observing a change in a homologous position. Under the simplest model, JC, the maximum likelihood estimator is

(1) $$\hat{t}=-\frac{3}{4}\ln(1-\frac{4}{3}d)\;.$$

#### 2.1.2 No strand-bias models

**Fig. 1.**
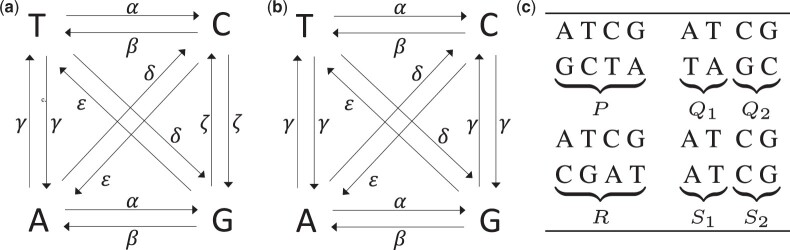
(**a**) Sueoka’s no strand-bias model of evolution with six rate parameters. TK5 model (**b**) is a special case of the six-parameter model with the constraint *r_AT_* = *r_GC_*. TK4 is the time-reversible version of the TK5 model with the condition $\omega =\frac{\beta }{\alpha +\beta }=\frac{\epsilon }{\epsilon +\delta }$ where *ω* is the total equilibrium frequency of bases *A* and *T*. (**c**) Nucleotide base pairs in homologous sites and their observed relative frequencies

A restriction of GTR, relevant to the study of next-generation sequencing (NGS) reads, is the model proposed by Sueoka (1995). Chargaff (1951) had earlier noted that in double-stranded DNA, the frequency of A should equal T, and that of G should equal C (parity rule 1). Thus, an i→j substitution occurring on the forward DNA strand must have an identical rate to an i→j substitution occurring on the reverse strand, which is the basis of Sueoka’s no strand-bias model (Fig. 1). Since an i→j entails an $\bar{i}\to \bar{j}$ substitution on its opposite strand, the model constrains $r_{ij}=r_{\bar{i}\bar{j}}$ and therefore reduces the number of independent parameters in the model to six. Surprisingly, the parity of A with T and C with G has been extensively documented on single-strand DNA as well (parity rule 2; Mitchell and Bridge, 2006). The reason behind parity on a single strand has been debated from the start (Forsdyke, 1995; Galtier and Lobry, 1997) and continues to be debated (Forsdyke, 2021; Meyer, 2021), with the two (not mutually exclusive) hypotheses based on (i) Sueoka’s model of mutational bias in the replication of polymerase in neutrally evolving genomes (Lobry, 1995; Sueoka, 1995) and (ii) Forsdyke’s structural model that invokes selective pressure. Regardless of the cause of parity rule 2, a no strand-bias model can be appropriate even for single-strand data, as Sueoka intended the model to be used.

In this article, we deal with conditions where the no strand-bias model is the *best* we can do due to parity rule 1. Assume that G is not a single-stranded sequence but a set of *n* homologous sequences $G_{1},G_{2},\ldots ,G_{n}$ (similarly for H) where each sequence $G_{i}$ or $H_{i}$ comes from an arbitrary strand. Inputs made of *k*-mers, reads or (unaligned) contigs can be viewed this way. With these data, *r_ij_* is unidentifiable from $r_{\bar{i}\bar{j}}$. The main limitation of the no strand-bias model is that it does not allow analytical calculation of distances (Zagordi and Lobry, 2005).

Predating Suoeka’s paper by 14 years, Takahata and Kimura (1981) introduced the five-parameter non-time reversible model TK5 (Fig. 1b) that imposes on the general six-parameter model the constraint $r_{AT}=r_{TA}=r_{GC}=r_{CG}=\gamma$ and assumes that $\pi_{A}=\pi_{T}=\omega /2$ and $\pi_{C}=\pi_{G}=(1-\omega )/2$. By imposing $\omega =\frac{\beta }{\alpha +\beta }=\frac{\epsilon }{\epsilon +\delta },$Takahata and Kimura (1981) introduce a time-reversible version of the TK5 model with four parameters, called TK4, and derive an analytical formula for distance estimation under TK4. This equation uses 16 combinations of bases possible at each site, as summarized in Figure 1c. Let *f_ij_* for i,j∈Σ denote the relative frequency of sites where the first and second genome has character *i* and *j*, respectively. We define $P=f_{AG}+f_{GA}+f_{TC}+f_{CT},\;Q=f_{AC}+f_{CA}+f_{TG}+f_{GT},R=f_{AT}+f_{TA},\;S=f_{CG}+f_{GC},\;S_{1}=f_{AA}+f_{TT}$, and $S_{2}=f_{CC}+f_{GG}$. Note that $P+Q_{1}+Q_{2}+R+S_{1}+S_{2}=1$. An unbiased estimated phylogenetic distance $\hat{t}$ between G and H is given by Takahata and Kimura. We note that the original article (Takahata and Kimura, 1981) has a mistake and has the term $\left(S_{1}+Q_{1}\right)$ instead of $\left(S_{1}-Q_{1}\right)$. Substituting the values of $X_{-}(T)$ and $Y_{-}(T)$, as defined in Equation (2) of the original paper, to Equation (18) in the original paper results in $\left(S_{1}-Q_{1}\right)$ instead of $\left(S_{1}+Q_{1}\right)$ and gives us the estimator:

(2) $$\begin{array}{c} \hat{t}=-\frac{1}{4}\ln[\left\{\frac{(S_{1}-Q_{1})(S_{2}-Q_{2})-{\left(\frac{P-R}{2}\right)}^{2}}{\omega (1-\omega )}\right\}\\ \cdot {\left\{1-\frac{P+R}{2\omega (1-\omega )}\right\}}^{8\omega (1-\omega )-1}], \end{array}$$

where *ω* can also be written as:

(3) $$\omega =S_{1}+Q_{1}+\frac{1}{2}(P+R)$$

**Fig. 2.**
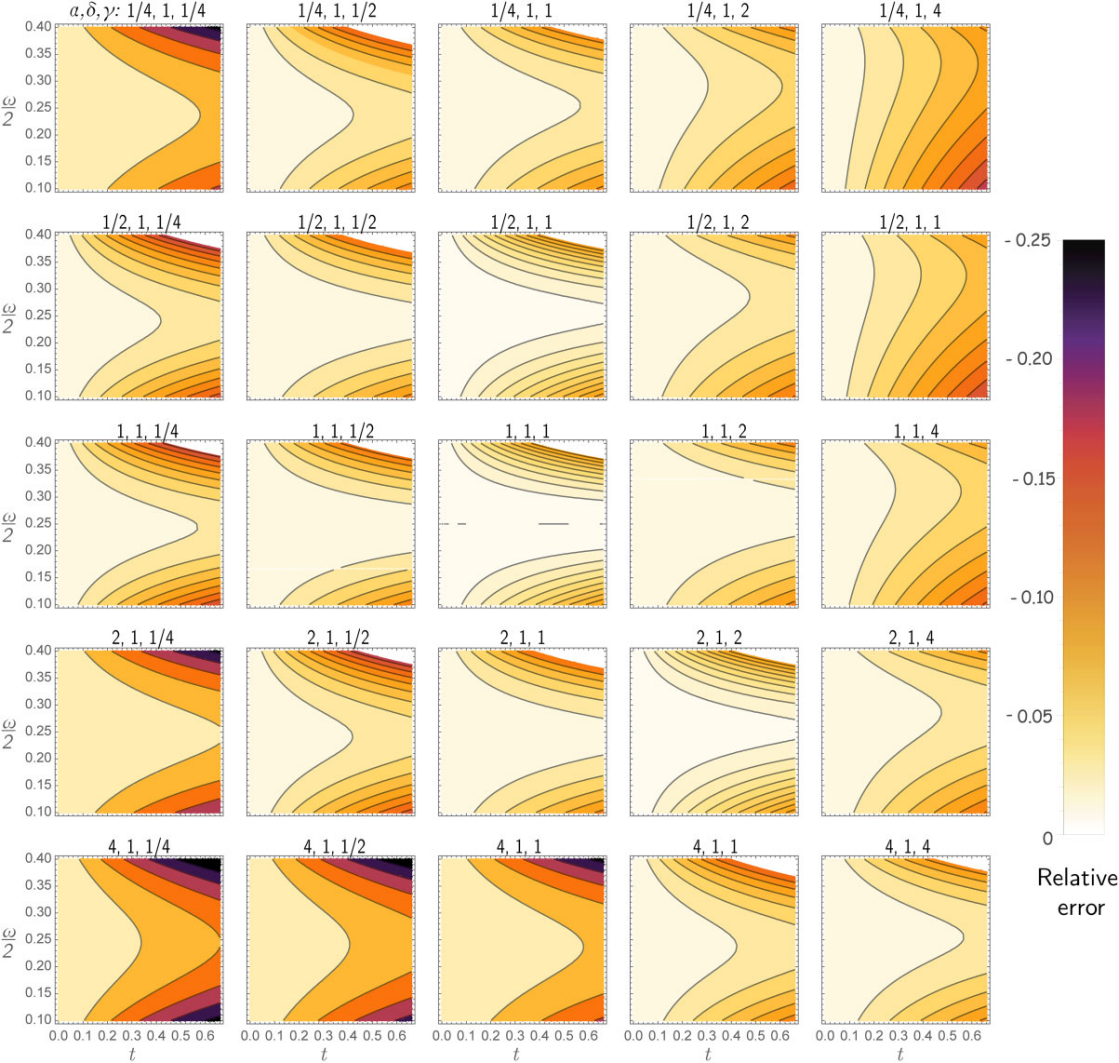
JC under-estimates TK4. The gradient shows relative bias of JC defined as $\frac{\hat{t}-t}{t}$ with $\hat{t}$ computed using Equation (1) where *d* is set to the expected hamming distance under TK4, which can be computed as $d=1-\pi .\text{diag}(e^{Rt})$. Each subplot corresponds to a choice of α,δ,γ, changing *α* across rows and *γ* across columns, fixing *δ *= 1. The *x*-axis changes the true evolutionary distance *t*, and the *y*-axis changes the base frequency parameter *ω*. Note that the JC model corresponds to α=δ=γ=4ω=1

Comparing (1) and (2), it is not obvious if the differences are consequential. By plotting the relative difference between (1) given the expected hamming distance under TK4 and the true time *t*, we can see that when parameters diverge from JC in biologically plausible ways, the often-used equation (1) can underestimate the true distance by more than 25% (Fig. 2). For example, with an AT-rich genome with ω=0.75, setting *α*  =  4 but keeping all other parameters equal to JC leads to 8% and 16% bias for true distances *t *=* *0.25 and 0.5, respectively. As expected, bias is reduced when TK4 parameters are all close to 1 (i.e. JC assumption). Overall, it seems that high levels of bias correspond to cases where some of the relative rates diverge from others while base frequencies also diverge substantially from 25% (both of which are biologically plausible).

#### 2.1.3 Assembly-free distance estimation

Although it is trivial to compute observed frequencies of substitutions between two aligned sequences, such calculations are challenging in the absence of alignment, for instance, when inputs are sets of unassembled reads. In the assembly-free setting, most methods assume the simple JC model, which only requires genomic distance. Luckily, various alignment-free methods can estimate *d* (Jain *et al.*, 2018; Ondov *et al.*, 2016; Sarmashghi *et al.*, 2019; Yi and Jin, 2013). Many of these algorithms (Sarmashghi *et al.*, 2019; Ondov *et al.*, 2016) break down the genome skims into *k*-mers.

We assume that a genome X is a finite i.i.d. stochastic process $X_{1}X_{2}\cdots X_{L}$ where each random variable (site) *X_m_* is drawn from categorical distribution with probability distribution $P[X_{m}=A]=P[X_{m}=T]=\pi_{A}=\pi_{T}=\omega /2$ and $P[X_{m}=C]=P[X_{m}=G]=\pi_{C}=\pi_{G}=(1-\omega )/2$. A *k*-mer at position *m* is $X_{m}X_{m+1}\cdots X_{m+k-1}$ and denoted with *x_m_* in short. We make the standard simplifying assumption of *k*-mer independence (*x_m_* is independent from all *k*−1 *k*-mers on each side). We denote the set of all *k*-mers in X with s(X). When *k* is sufficiently large with respect to *L* and *ω*, we can assume that |s(X)|≈L. A second genome Y originates from X through a substitution process described earlier. The probability of a match between two homologous *k*-mers is ${\left(1-d\right)}^{k}$. Therefore, the expected total number of homologous *k*-mer matches between s(X) and s(Y) is approximately $S=L\cdot {\left(1-d\right)}^{k}$ (Fan *et al.*, 2015; Ondov *et al.*, 2016; Sarmashghi *et al.*, 2019). Denoting by C=SL the containment Jaccard index, note that

(4) $$\hat{d}=1-C^{\frac{1}{k}}\;.$$

The *Jaccard index J*, defined as the intersection divided by the union of two sets, is easy to compute using techniques such as min-hash (Ondov *et al.*, 2016). Thus, instead of *C*, most methods have relied on *J*, which is intimately connected to *C* because $J=\frac{S}{2L-S}$ and thus, $C=\frac{S}{L}=\frac{2J}{1+J}$ Finally, following the TK4 notations, $\hat{d}=P+Q_{1}+Q_{2}+R\;$ holds.

### 2.2 Containment Jaccard correction

In addition to homologous ones, *k*-mers in non-homologous positions in the two genomes can also match, albeit with lower probability. Distance estimation using the Jaccard index requires computing the number of shared *k*-mers through homology. The number of non-homologous *k*-mer matches contributing to |s(X)∩s(Y)| is negligible in most settings when *k* is large enough for the size of the alphabet; e.g. *k *=* *31 with |Σ|=4, leading to $4^{31}\approx 4\times 10^{18}$ possible *k*-mers. However, our algorithm for estimating TK4 distances requires reducing the alphabet set to three letters, which may lead to biased probabilities based on the value of *ω*. Under such conditions, the non-homologous *k*-mer matches cannot be ignored.

Röhling *et al.* (2020) have derived an expression for the expected number of *k*-mers *x_m_* and *y_n_*, n≠m that match between the two genomes by chance (i.e. not through homology). However, to compute the contribution of non-homologous *k*-mer matches to |s(X)∩s(Y)|, not only we need to know the expected number of *k*-mers matching by chance, we also need to account for a *k*-mer *x_m_* matching multiple *k*-mers in the other genome. Consequently, we propose a more precise estimate for the cardinality of the intersection between two random genomes.

Lemma 1 *The expected value of* $\tilde{C}=\frac{\left|s(X)\cap s(Z)\right|}{L}$*, containment Jaccard for k-mers between two genomes* X*and* Z*generated by two i.i.d processes with stationary distribution* $\pi_{A}=\pi_{T}=\omega /2$*and* $\pi_{C}=\pi_{G}=\frac{1-\omega }{2}$*, is:*

(5) $$\begin{array}{c} E[\tilde{C}]=\frac{2^{k}}{L}\sum_{a=0}^{k}{\left(1-{\left(1-{\left(\frac{\omega }{2}\right)}^{a}{\left(\frac{1-\omega }{2}\right)}^{k-a}\right)}^{L}\right)}^{2}\left(\begin{array}{c} k\\ a \end{array}\right) \end{array}.$$

*Proof*. For 0≤a≤k, let $r\in \Sigma^{k}$ be a *k*-mer with *a* A’s and T’s.

$$P(x_{m}=r)=P(z_{m}=r)={\left(\frac{w}{2}\right)}^{a}{\left(\frac{1-w}{2}\right)}^{k-a}\;.$$

Thus, due to the independence assumption of *x_m_* from its overlapping neighbors, the probability of *r* being in set s(X) is the following

$$\begin{array}{l} P(r\in s(X))=1-P(r\notin s(X))=1-\prod_{m=1}^{L}P(r\neq x_{m})\\ =1-{\left(1-P(r=x_{m})\right)}^{L}\\ =1-{\left(1-{\left(\frac{w}{2}\right)}^{a}{\left(\frac{1-w}{2}\right)}^{k-a}\right)}^{L} \end{array}.$$

Results follow by noting that there are $2^{k}\left(\begin{array}{c} k\\ a \end{array}\right)$ many selections for each *r* and P(r∈s(X))=P(r∈s(Z)). □

By stationarity of the substitution process, Y has the same base frequencies as X. Thus, |s(X)∩s(Z)| can be used to estimate the non-homologous portion of |s(X)∩s(Y)|. In other words, |s(X)∩s(Y)|−|s(X)∩s(Z)| is the number of homologous *k*-mers. Combining (4) and (5), *d* can be estimated from the containment Jaccard *C* of X and Y:

(6) $$\hat{d}=1-{\left(C-E[\tilde{C}]\right)}^{\frac{1}{k}}.$$

On unassembled data, we account for lack of coverage and sequencing errors when computing $\hat{d}$ using the approach of Skmer (Sarmashghi *et al.*, 2019) as detailed in Supplementary Methods.

### 2.3 Calculation of TK4 terms via replacement

Given the possibility of high error with the JC model (Fig. 2), we would like to develop alignment-free methods of computing distances according to the TK4 model using (2). Therefore, our goal is to *estimate* the terms *P*, *Q*_1_, *Q*_2_, *R*, *S*_1_, *S*_2_ and *ω*. Consider the replacement technique where every occurrence of a character i∈Σ in X and Y is replaced with character j∈Σ, i≠j. Let *d_ij_* be the genomic distance between two genomes after such replacement. The reduction in genomic distance after *i* to *j* substitution is exactly $f_{ij}+f_{ji}$. Using the Equation (6), *d_ij_* can be estimated from empirical containment Jaccard $C_{ij}$ and expected number of background *k*-mer matches $E[{\tilde{C}}_{ij}]$. Using this replacement scheme, the *P*, *Q*_1_, *Q*_2_ and *R* terms in (2) are estimated as follows:

(7) $$\begin{array}{c} P=2\hat{d}-{\hat{d}}_{AG}-{\hat{d}}_{CT}\;\;Q_{1}=\hat{d}-{\hat{d}}_{AT}\\ R=2\hat{d}-{\hat{d}}_{AC}-{\hat{d}}_{GT}\;\;Q_{2}=\hat{d}-{\hat{d}}_{CG} \end{array}.$$

As base frequencies $\omega =\frac{\left(\pi_{A}+\pi_{T}\right)}{2}$ can be trivially computed from X and Y, we can compute the remaining terms *S*_1_ and *S*_2_ using (3):

(8) $$\begin{array}{c} S_{1}=\omega -Q_{1}-\frac{P+R}{2}\;\;S_{2}=1-\omega -Q_{2}-\frac{P+R}{2} \end{array}.$$

As mentioned previously, estimating *d_ij_* requires computation of $E[{\tilde{C}}_{ij}]$. Calculating this term depends on the type of replacement. Lemma 1 can be easily updated to account for replacements. For instance,

(9) $$\begin{array}{c} E[{\tilde{C}}_{AT}]=\frac{1}{L}\sum_{a=0}^{k}{\left(1-{\left(1-\omega^{a}{\left(\frac{1-\omega }{2}\right)}^{k-a}\right)}^{L}\right)}^{2}\left(\begin{array}{c} k\\ a \end{array}\right)2^{k-a}\\ E[{\tilde{C}}_{CG}]=\frac{1}{L}\sum_{a=0}^{k}{\left(1-{\left(1-{\left(\frac{\omega }{2}\right)}^{a}{\left(1-\omega \right)}^{k-a}\right)}^{L}\right)}^{2}\left(\begin{array}{c} k\\ a \end{array}\right)2^{a}\\ E[{\tilde{C}}_{AC}]=E[{\tilde{C}}_{AG}]=\\ \frac{1}{L}\sum_{a=0}^{k}\sum_{b=0}^{k-a}{\left(1-{\left(1-{\left(\frac{1}{2}\right)}^{a}{\left(\frac{\omega }{2}\right)}^{b}{\left(\frac{1-\omega }{2}\right)}^{k-a-b}\right)}^{L}\right)}^{2}\left(\begin{array}{c} k\\ a \end{array}\right)\left(\begin{array}{c} k-a\\ b \end{array}\right) \end{array}.$$

Since letter replacements (especially A to T for ω>0.5 and G to C for ω<0.5) lead to a high expected number of shared *k*-mers by chance, correcting for random matches is essential. For example, with a pair of genomes of length 10^8^ and ω=0.6, the expected number of background matches between two-way genomes after A-to-T replacement is 289 000, which is 5× larger than the number of homologous *k*-mer matches when *t *=* *0.5. Supplementary Figure S2 shows the accuracy of Equation (9) and their improvement over simply using the expected number of *k*-mer matches derived by Röhling *et al.* (2020).

### 2.4 Handling mixed-strand conditions

We now consider the case in which each k-mer in X and Y may come from the forward or reverse DNA strand arbitrarily. In practice, chromosomes or contigs in an assembly or reads in a sequencing run may come arbitrarily from either forward or reverse strands. For simpler exposition, assume each genome consists of a single contig from an unknown strand (the method can handle any number of contigs or reads). Let X′ be another finite i.i.d. stochastic process $X_{1}^{\prime }X_{2}^{\prime }X_{3}^{\prime }\ldots X_{L}^{\prime }$ such that is $X_{i}^{\prime }=X_{i}$ with some unknown but fixed probability $p_{x}>0$ and $X_{i}^{\prime }={\bar{X}}_{L-i}$ with probability $1-p_{x}$ where ${\bar{X}}_{i}$ is the reverse complement (RC) of *X_i_*. Y′ is defined similarly. The genomic distance between X and Y can still be computed using (4) by using canonical k-mers, a concept utilized by several tools (Marçais and Kingsford, 2011; Ondov *et al.*, 2016). We utilize the same concept and construct a two-way genome $Z^{˙}=Z_{1}^{\prime }Z_{2}^{\prime }Z_{3}^{\prime }\ldots Z_{L}^{\prime }{\bar{Z}}_{L}^{\prime }{\bar{Z}}_{L-1}^{\prime }{\bar{Z}}_{L-2}^{\prime }\ldots {\bar{Z}}_{1}^{\prime }$ with Z∈{X,Y} by adding the RC of each genome to itself. By design, both forward and reverse copies of each k-mer in Z are present in $Z^{˙}$. If *x_m_* = *y_m_*, either $\left({\dot{x}}_{m}={\dot{y}}_{m})\wedge ({\dot{x}}_{2L-m}={\dot{y}}_{2L-m}\right)$ or $\left({\dot{x}}_{m}={\dot{y}}_{2L-m})\wedge ({\dot{x}}_{2L-m}={\dot{y}}_{m}\right)$. Either way, the number of homologous *k*-mer matches and genome length both double compared to the case where all sequences are of the same strand, leaving containment Jaccard due to homologous *k*-mers unchanged; thus, Equation (6) is applicable to two-way genomes as long as $E[\tilde{C}]$ is computed with 2 *L*.

Similarly to the replacement technique, we introduce *i* to *j* replacements on a two-way genome. For each homologous site (*X_m_*, *Y_m_*) in the base genomes X and Y, we have two pairs of homologous sites in $X^{˙}$ and $Y^{˙}$. Although there are four alternative choices for assignment of forward and reverse strand to $\left\{{\dot{X}}_{m},{\dot{Y}}_{m},{\dot{X}}_{2L-m},{\dot{Y}}_{2L-m}\right\}$, without loss of generality, let $\left({\dot{X}}_{m},{\dot{Y}}_{m})=(X_{m},Y_{m}\right)$ and $\left({\dot{X}}_{2L-m},{\dot{Y}}_{2L-m})=({\bar{X}}_{m},{\bar{Y}}_{m}\right)$. After replacing every occurrence of *i* with *j* in $X^{˙}$ and $Y^{˙}$,

$$\begin{array}{c} P({\dot{X}}_{m}={\dot{Y}}_{m})=1-(d-P(X_{m}=i,Y_{m}=j)\\ -P(X_{m}=j,Y_{m}=i))\\ P({\dot{X}}_{2L-m}={\dot{Y}}_{2L-m})=1-(d-P(X_{m}=\bar{i},Y_{m}=\bar{j})\\ -P(X_{m}=\bar{j},Y_{m}=\bar{i})) \end{array}.$$

The reduction in the genomic distance between $X^{˙}$ and $Y^{˙}$ after the replacement, $\hat{d}-{\hat{d}}_{ij}$, is $f_{ij}+f_{ji}$ in the forward strand (i.e. $\left({\dot{X}}_{1},{\dot{Y}}_{1})\ldots ({\dot{X}}_{L},{\dot{Y}}_{L}\right)$) and $f_{\bar{i}\bar{j}}+f_{\bar{j}\bar{i}}$ in the reverse strand (i.e. $\left({\dot{X}}_{L+1},{\dot{Y}}_{L+1})\ldots ({\dot{X}}_{2L},{\dot{Y}}_{2L}\right)$). The overall reduction is the average of the reduction in the forward and reverse strands, which is $\frac{1}{2}(f_{ij}+f_{ji}+f_{\bar{i}\bar{j}}+f_{\bar{j}\bar{i}})$. As a result, ${\hat{d}}_{AG}={\hat{d}}_{CT}$ and ${\hat{d}}_{AC}={\hat{d}}_{GT}$. The *P*, *Q*_1_, *Q*_2_ and *R* terms in (2) are estimated from two-way genome using:

$$\begin{array}{c} P=2\hat{d}-2{\hat{d}}_{AG}\;\;Q_{1}=\hat{d}-{\hat{d}}_{AT}\\ R=2\hat{d}-2{\hat{d}}_{AC}\;\;Q_{2}=\hat{d}-{\hat{d}}_{CG} \end{array}.$$

Thus, we need to compute only five values from the data, $\hat{d},\;{\hat{d}}_{AC},\;{\hat{d}}_{AG},\;{\hat{d}}_{AT}$ and ${\hat{d}}_{CG}$ in addition to an estimation of *ω*.

Although *P*, *Q*_1_, *Q*_2_ and *R* can be determined independently given the estimate $\hat{d}$, they must satisfy the constraint $P+Q_{1}+Q_{2}+R=\hat{d}$. Thus, $\hat{d}=2\hat{d}-2{\hat{d}}_{AG}+\hat{d}-{\hat{d}}_{AT}+\hat{d}-{\hat{d}}_{CG}+2\hat{d}-2{\hat{d}}_{AC}$. Since all five estimated values $\hat{d},\;{\hat{d}}_{AC},\;{\hat{d}}_{AG},\;{\hat{d}}_{AT}$ and ${\hat{d}}_{CG}$ are empirical, it cannot be ensured that this equation will be satisfied. In other words, the system of equations has one excess observation. Among the five, the distance with no replacements $\hat{d}$ is always the largest, i.e. has the lowest containment Jaccard index. For large distances, the containment Jaccard can be zero, which prohibits computing any evolutionary distance (JC or TK4) from the data. In order to increase the distance upper-bound of *TK*4 model, we opt to reduce the number of free variables in the system by computing $\hat{d}$ from ${\hat{d}}_{ij}$, not directly from data. More precisely,

(10) $$\begin{array}{c} \hat{d}=2\hat{d}-2{\hat{d}}_{AG}+\hat{d}-{\hat{d}}_{AT}+\hat{d}-{\hat{d}}_{CG}+2\hat{d}-2{\hat{d}}_{AC}\\ =(2{\hat{d}}_{AG}+2{\hat{d}}_{AC}+{\hat{d}}_{AT}+{\hat{d}}_{CG})/5 \end{array}.$$

We use this equation to compute JC model distances using (1) and to calculate *P*, *Q*_1_, *Q*_1_ and *R* as a linear combination of four ${\hat{d}}_{ij}$ distances calculated using (6) after replacement:

(11) $$\begin{array}{c} P=(-6{\hat{d}}_{AG}+4{\hat{d}}_{AC}+2{\hat{d}}_{AT}+2{\hat{d}}_{CG})/5\\ R=(4{\hat{d}}_{AG}-6{\hat{d}}_{AC}+2{\hat{d}}_{AT}+2{\hat{d}}_{CG})/5\\ Q_{1}=(2{\hat{d}}_{AG}+2{\hat{d}}_{AC}-4{\hat{d}}_{AT}+{\hat{d}}_{CG})/5\\ Q_{2}=(2{\hat{d}}_{AG}+2{\hat{d}}_{AC}+{\hat{d}}_{AT}-4{\hat{d}}_{CG})/5 \end{array}.$$

### 2.5 NSB: TK4 distance estimation using *k*-mers

Algorithm 1 combines results in the previous sections into a three-step process (Supplementary Fig. S1) for estimating phylogenetic distances under the TK4 model. We implemented the algorithm using Python in a method called the NSB (No Strand-Bias) distance estimator. In its first step, NSB adds the RC of all input sequences. It then builds separate *k*-mer libraries for each of the inputs using a left/right encoding scheme where nucleotide bases *A*, *C*, *G* and *T* are represented as two-bit numbers, thus requiring 64-bit integer for *k* ≤ 32. NSB then builds base substituted encoded *k*-mer libraries from the initial encoded library by replacing the encoded bits of base *i* with the encoded bits of base *j*, for (i,j)∈{(A,C),(A,G),(A,T),(C,G)}. Thanks to a Left/Right encoding scheme, a replacement operation on an array of *k*-mers can be computed rapidly using fast and vectorized bitwise operations such as XOR, AND and Shift (e.g. see A_to_C function in Algorithm 1). Finally, NSB computes the Jaccard indices for four pairs of base-substituted encoded libraries by computing the cardinality of the intersection succeeded by containment Jaccard correction. In practice, input genomes are seldom the same size and with the same base frequencies. When computing $E[{\tilde{C}}_{ij}]$ using Lemma 1, P(r∈s(X)) and P(r∈s(Y)) are computed using *L* and *ω* of the respective genome for a given *k*-mer *r*. In the final stage, we estimate the phylogenetic distance of each pair of genomes using Equation (2). Various components in this equation are calculated using the Equations (8)–(11). *L* and *ω* are set to the average of the two input genomes. When input data are unassembled (reads), we run Skmer prior to NSB to obtain *L*, coverage and sequencing error rate. Computing the cardinality of the intersection between two encodings of size *N* takes O(N log ⁡(N)) time and *O*(*N*) memory. Therefore, the time and memory complexity of Algorithm 1 are $O(n^{2}N\;\text{log}\;(N))$ and *O*(*N*) since no more than two encodings are loaded into the memory simultaneously.

Algorithm 1. NSB: TK4 Distance estimation. We denote the set of all reference sequences by *S*. NSB first runs PREPROCESS; ADD_RC computes the RC of a genome. It then calculates pairwise distances of the sequences according to the PAIRWISE-DIST procedure. BG_INTERSECT computes expected number of background matches after replacement the using Equation (9).

1: **procedure** PREPROCESS(S)

2:  **for**G∈S**do**

3:   E←ENCODE(ADD_RC(*G*))

4:   **for**(i,j)∈{(A,C),(A,G),(A,T),(C,G)}**do**

5:    $E_{ij}\leftarrow \emptyset$

6:    **for**e∈E**do**

7:      $E_{ij}\leftarrow E_{ij}\cup \{$ i_TO_j(e) }

8:   Save $\left\{E_{AC},E_{AG},E_{AT},E_{CG}\right\}$ to disk

9: **procedure** ENCODE($G_{2way}$)

10:  E←∅

11:  **for** *k*-mer $a\in \;G_{2way}$**do**

12:   e←2k bit zeros

13:   **for** letter $l_{i}\in a$**do**

14:    $e_{i}\leftarrow 1$ if $l_{i}\in \{C,G\}$

15:    $e_{i+k}\leftarrow 1$ if $l_{i}\in \{A,G\}$

16:   E←E∪{e}

17:  **return** *E*

18: **procedure** A_TO_C(*e*) ▹ an example of i_TO_j function

19:  $\mathrm{mask}\leftarrow 2^{k}-1$

20:  $e_{1}\leftarrow$ first *k* bits of *e*

21:  $e_{2}\leftarrow$ last *k* bits of *e*

22:  $e_{3}\leftarrow e_{2}$ & (e1 ⊕ mask)

23:  $e_{1}\leftarrow e_{1}\;⊕\;e_{3}$

24:  $e_{2}\leftarrow e_{2}\;⊕\;e_{3}$

25:  **return** 2*k* bits $\left((e_{1}\ll k)+e_{2}\right)$

26: **procedure** PAIRWISE-DIST(*G*_1_, *G*_2_)

27:  **for**(i,j)∈{(A,C),(A,G),(A,T),(C,G)}**do**

28:   $(E_{ij,1},E_{ij,2})\leftarrow$ Read encoded (*G*_1_, *G*_2_) from disk

29:   $D_{ij}\leftarrow$ GENOME_DIST($E_{ij,1},E_{ij,2},L_{1},L_{2},\omega_{1},\omega_{2}$)

30:  **return** CLC-TK4-DIST($D_{AC},D_{AG},D_{AT},D_{CG}$)

31: **procedure** CLC-TK4-DIST($D_{AC},D_{AG},D_{AT},D_{CG}$)

32:  $D\leftarrow (2D_{AG}+2D_{AC}+D_{AT}+D_{CG})/5$

33:  $P\leftarrow D-D_{AG}$

34:  $Q_{1}\leftarrow D-D_{AT}$

35:  $Q_{2}\leftarrow D-D_{CG}$

36:  $R\leftarrow D-D_{AC}$

37:  $\omega \leftarrow (\omega_{1}+\omega_{2})/2$

38:  $S_{1}\leftarrow \omega -(P+R)/2-Q1$

39:  $S_{2}\leftarrow 1-\omega -(P+R)/2-Q2$

40:  **return** TK4 distance using Equation (2)

41: **procedure** GENOME_DIST($E_{ij,1},E_{ij,2},L_{1},L_{2},\omega_{1},\omega_{2}$)

42:  $I\leftarrow |E_{ij,1}\cap E_{ij,2}|$

43:  $I_{c}\leftarrow$ BG_INTERSECT($i,j,L_{1},L_{2},\omega_{1},\omega_{2}$)

44:  $C\leftarrow 2(I-I_{c})/(L_{1}+L_{2})$ # Containment Jaccard

45:  **return**$1-{\left(C\right)}^{\frac{1}{k}}$

## 3 Validation results

We validate NSB in simulations and on real data and compare it to three methods. NSB-JC is JC distance computed using (10) and (1) with our tool. We also test using Jellyfish (2.3.0) and Skmer (3.1.0) to estimate containment Jaccard index and subsequently JC distance using (1) and (6). Jellyfish computes Jaccard exactly, and Skmer approximates it using 10^5^ sketches. On genome skims, we compare NSB-TK4 to Skmer.

### 3.1 Simulation study

#### 3.1.1 Simulating genome sequences under the TK4 model

We use our own procedure to simulate pairs of genomes evolved under the TK4 model with controlled levels of distance and model parameters (https://github.com/balabanmetin/tk4-evol-sim). First, we either use a real genome as the ancestral genome or simulate one by drawing each site randomly from *π* with user-defined *ω*. We simulate two separate genomes from the ancestral genome by introducing substitutions at random positions. The frequency of each substitution type is determined by the TK4 model transition probability matrix **P** and half of the targeted distance t/2, producing two genomes with the evolutionary distance *t*. We create two simulated datasets. The first dataset uses a randomly generated 100 Mb base genome with ω=0.6. The second dataset uses a real assembled genome of *Saccharomyces arboricola* (11 Mb) as the base sequence. The base frequencies of the available *S.arboricola* genome are $\pi_{A}\approx \pi_{T}\approx 0.307$ and $\pi_{C}\approx \pi_{G}\approx 0.193$, which follow the assumptions of TK4 with ω=0.614. We set the parameters of the TK4 model according to Figure 1, exploring eight values of *α*, *δ*, and *γ*. Recall that δα=ϵβ and $\omega =\frac{\beta }{\beta +\alpha }=\frac{\epsilon }{\epsilon +\delta }$, leaving us with only three free parameters for a fixed *w*. We generated eight model conditions with different TK4 parameters (Supplementary Table S1) chosen to include cases with both minimal and substantial deviations from the JC model based on the earlier calculations (Fig. 2). For each model condition, we simulated genome sequences with true distances t∈{0.01,0.05,0.1,0.2,0.3,0.4,0.5}, each with 10 replicates, covering a range of both short and long distances.

#### 3.1.2 Results on simulations under the TK4 model

##### 3.1.2.1 Random base genomes

**Fig. 3.**
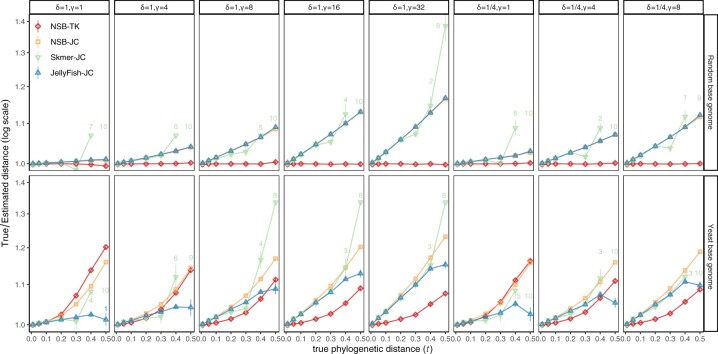
Comparing the accuracy of distances estimated by different approaches on random and Yeast-based simulated genomes. Genome sequences were simulated by randomly substituting the genome skims of *S.arboricola* (11 Mb) and a random 100 Mb sequence with eight sets of TK4 parameters and with seven controlled true distances. Here, *ω* is fixed, and since these rates do not have a scale, *α *= 1 in all cases. We show the average true distance divided by estimated distances (*y*-axis) with standard errors (over replicates, requiring at least two) against the true distances. Annotated numbers show the number of replicates out of 10 where Skmer or JellyFish return infinity. See Supplementary Figure S4 for linear scale

When input genomes are generated in the i.i.d. fashion assumed by both evolutionary models, across all model conditions, and regardless of the true phylogenetic distances *t*, the distances estimated by NSB-TK4 are highly accurate (Fig. 3). By contrast, JC distances are accurate when the true distance *t* is low but are under-estimated when *t* increases. In the most challenging case, *t *=* *0.5, NSB-TK4 deviates only 0.3% from the true value on average compared to 7.8% for Jellyfish-JC. The error of Jellyfish-JC is as high as 18% when *γ* = 32, which causes extreme deviations from JC. The best performance of JC is when all parameters except *ω* follow JC. As models become successively more deviant from JC assumptions, the accuracy of JC diminishes.

Finally, comparing the two ways of obtaining JC distances, for t≤0.3, the approximate Skmer distances are slightly *more* accurate than Jellyfish. However, when *t *>* *0.3, Skmer distances become less accurate. When true distance t≥0.4, Skmer fails to estimate distances in some cases (most cases for *t *=* *0.5) because the true Jaccard index becomes too small (e.g. $<10^{-5}$) to compute reliably with sketches of size 10^5^.

##### 3.1.2.2 Yeast-base simulations

The TK4-based calculations show improvements over JC computed using NSB or Skmer across some model conditions except for δ=γ=1 that resembles JC (Fig. 3). However, the comparison to JC computed exactly (using JellyFish) is more complex. When deviations from JC are relatively low, JellyFish-JC can be as accurate or even more accurate than NSB-TK4. It is only with higher levels of deviation from JC that improvements of NSB-TK4 over JC are clear. Regardless of simulation parameters, phylogenetic distances t≤0.1 are estimated with high accuracy under both TK4 and JC models. However, the JC model starts to underestimate the distance as we increase the distance *t*, and the underestimations are substantial when t≥0.3. Moreover, the JC error is not linear or even monotonically increasing with *t*, meaning that the distance matrices obtained from the JC model may not be additive. When *t* is increased to 0.5, TK4-based distances tend to have reasonable accuracy with a few exceptions (e.g. for *γ *= 8). In some cases, TK4 distances have more than 10% error with increased *t* and are consistently less accurate in three conditions than JC. Comparing the results to random base genomes, the reduced accuracy of TK4 on these conditions has to be due to violations of the model in the base genome, a point that we will return to in the Discussion section.

Finally, we explore the impact of the choice of *k*-mer size on accuracy. We select the simulated yeast genomes with a fixed model-condition *δ* = 1 and *γ* = 4 and test k∈{21,23,25,27,29,31} over 10 replicates. We do not explore *k *>* *31 because Jellyfish and NSB do not support it. No single *k* value performs universally better than others (Supplementary Fig. S3); the choice depends on the distance and the method. For *k *=* *21, NSB-TK4 overestimates or underestimates the true distance when d≤0.1 or 0.1<d≤0.4, respectively. On the other hand, for *d* values larger than 0.4, NSB-TK4 does not return a valid distance due to overestimation of the number of background matches. As *k* increases, distance estimation using NSB-TK4 becomes more accurate, reaching peak accuracy with *k *=* *31. More generally, NSB-TK4 and NSB-JC are more sensitive to the selection of *k* than Jellyfish and Skmer. For example, when *d *=* *0.4, the estimation error difference between the most and the least accurate estimates are 13.7% (*k *=* *31 and *k *=* *23), 12.5% (*k *=* *31 and *k *=* *23), 6.3% (*k *=* *27 and *k *=* *31) and 1.2% (*k *=* *31 and *k *=* *21) for NSB-TK4, NSB-JC, Skmer-JC and Jellyfish-JC, respectively. Given the totality of results, we recommend setting *k *=* *31 for NSB-TK4.

#### 3.1.3 Simulation of phylogenies under the GTR model

To compare TK4 and JC models under the presence of model misspecification, we simulate an eight-taxa dataset with genomes evolved under the GTR model (Tavaré, 1986), which can substantially violate the assumptions of JC and TK4 models. Of the 120 fully balanced and caterpillar tree topologies simulated by Rachtman *et al.* (2022) using Simphy (Mallo *et al.*, 2016), we first proceed with taking the first 20 for each category. In these eight-taxa trees, branch lengths are randomly selected from the log-uniform distribution ranging between 0.00001 and 0.12. Next, we simulate 10 Mb genome sequences using INDELible (Fletcher and Yang, 2009). Base frequences of the GTR model follow $\{\pi_{A},\pi_{C},\pi_{G},\pi_{T}\}=\{\omega /2,\left(1-\omega \right)/2,\left(1-\omega \right)/2,\omega /2\}$ where *ω* is a drawn from *Beta* (30, 21) distribution. Other entries of the GTR matrices are drawn from Dirichlet distribution with parameters (50,7,12,12,14,50) corresponding to C↔T, A↔T, G↔T, A↔C, G↔C, G↔A. Each method produces an 8 × 8 distance matrix, which is then given to FastME 2.0 (Lefort *et al.*, 2015) to estimate the phylogeny. Since we have a tree, we compare the methods by measuring Robinson–Foulds (RF) (Robinson and Foulds, 1981) distance between the true tree and the inferred tree. Beyond topological accuracy, we quantify the divergence of the TK4 and JC distances from the additivity using the FME (Fitch and Margoliash, 1967) weighted least squares error. Since FME metric weights distances by ${\hat{t}}^{-2}$, it is insensitive to the unit and scale of branch lengths. When measuring the FM metric, we use the combination of true tree topology and estimated distances, which ensures measurements across different methods are based on the same (true) tree.

#### 3.1.4 Results on phylogenies evolved under the GTR model

**Fig. 4.**
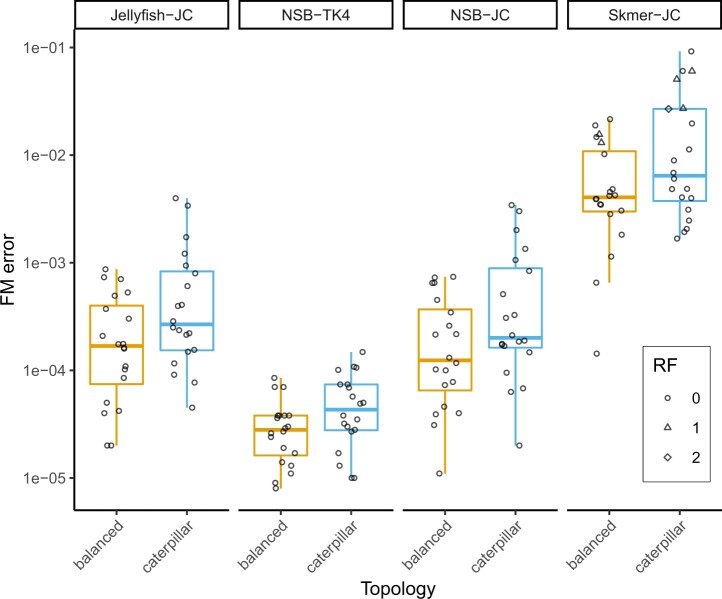
Deviation from additivity measured for TK4 and JC models on the dataset of 40 eight-taxa phylogenies simulated under the GTR model. The dataset consists of 20 balanced and 20 caterpillar trees. Whiskers in the boxplot demonstrate the range between the first and third quartiles. Point shape represents the RF distance between the constraint-free tree inferred by the method and the true tree. *Y*-axis is in log-scale

Topological accuracy remains high even with model mis-specification (Fig. 4). NSB-TK4, NSB-JC and JellyFish-JC infer the correct topology in all 40 cases, whereas Skmer-JC is erroneous in 6/40 trees tested. The mean FME error of NSB-TK4 (4e-05) is an order of magnitude lower than those of NSB-JC and Jellyfish-JC (5e-04), which have near identical levels of error. Therefore, in simulations, the TK4 model produces distances closer to additivity than JC when model misspecification is present. However, Skmer-JC has 27 times higher error than the other two JC-based methods, indicating that the sketching process affects accurate distance estimation to a higher degree than model misspecification. Finally, regardless of the method used, the 20 replicates with balanced topologies tend to have lower deviations from additivity than those based on unbalanced topologies.

### 3.2 Evaluation on biological bacterial data

We created a dataset consisting of 10 clades of microbial species subsampled from the Web of Life (WoL; Zhu *et al.*, 2019) ASTRAL tree of 10575 Bacteria and Archaea taxa. We started by finding all the clades with 30–50 leaves and 0.2–0.7 diameter (the maximum pairwise tree distance between any pair). We then selected the top 25 clades with the highest support and for each clade, computed an all-pairwise distance matrix using Skmer (sketch size 10 million), inferred a phylogenetic tree using FastME 2.0, and computed the RF distance between the WoL ASTRAL reference tree and the inferred tree. We then selected nine clades with the lowest RF distance, and these clades had 32–46 species and RF distances between 0.16 and 0.42. As none of the nine selected clades had any missing data in their distance matrix, we also curated a challenging subtree with 86 taxa from the *Erysipelotrichaceae* family from the WoL tree that contained 114 missing data entries in its distance matrix (RF distance: 0.43) computed using Skmer.

#### 3.2.1 Results on bacterial dataset

**Fig. 5.**
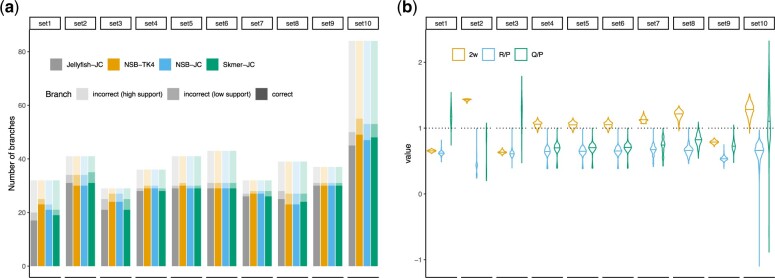
(**a**) Comparison of different methods to the ASTRAL tree on 10 subsets of the bacterial dataset. We show the number of branches in the reference tree that are correctly estimated or are incorrectly estimated and have low (less than 0.95) or high support in the reference tree. (**b**) TK4 model parameters inferred using NSB-TK4 for each set. Deviations from *y *=* *1 indicate violation of the JC model. $Q=Q_{1}+Q_{2}$

On the 10 bacterial datasets, while methods are generally competitive (Fig. 5a), overall, NSB-TK4 is better than others as it produces the best result in 8 datasets out of 10. The total number of missing branches for NSB is 120 (out of 403; Supplementary Table S2), which is lower than Jellyfish, with 133 missing branches. Results are similar when focusing on highly supported branches: NSB-TK4 misses 95 out of 374 branches with at least 0.95 support, while Jellyfish misses 109. Among the three methods that compute JC distances, NSB-JC is the most accurate, matching or improving on Jellyfish and Skmer in 7 out of 10 cases and with eight and four fewer wrong branches, respectively. In the most challenging case (Set 10), the distance matrix produced by NSB-TK4 contains 20 fewer missing entries (infinity) than both Jellyfish-JC and Skmer-JC. As a result of its replacement technique, NSB can compute distances where other tools cannot. To perform a tree inference on distance matrices with missing data, we impute the missing distances using a machine-learning-based algorithm (Bhattacharjee and Bayzid, 2020). Here, NSB-TK4 distances produce the tree with the fewest differences to the reference phylogeny compared to JC-based tools.

Jellyfish-JC had between 7% and 57% (mean: 22%) higher FM error than NSB-TK4 across datasets (Supplementary Table S3). NSB-TK4 distances are not only more additive but also on average 13% and 32% larger than those of Jellyfish and Skmer, which may underestimate the distances.

TK4 model parameters inferred by NSB-TK4 demonstrate that JC model assumptions are significantly violated in the real data (Fig. 5b). For instance, 2ω, assumed to be 1 in the JC model, is as low as 0.65 on average across all pairs in a set. In addition, transversion to transition ratios *R*/*P* and $(Q_{1}+Q_{2})/P$ are less than 1 in almost every case, in clear violation of the JC model; thus, NSB captures the long understood (Yang and Yoder, 1999) divergence of transversion and transition rates.

### 3.3 Evaluation on biological yeast dataset

We also study an existing the yeast dataset used (Balaban and Mirarab, 2020), consisting of eight genomes (Supplementary Table S4) with sizes in the 10.9–12.4 Mb range and the number of scaffolds varying between 16 and 2808. We use ART v2.5.8 (Huang *et al.*, 2012) to create *in silico* genome skims of 150 bp reads with Illumina HiSeq 2500 error profile. We test for 1, 2, 4 and 8× sequencing coverage levels. We use a published yeast phylogeny (Shen *et al.*, 2016) as the reference and compare it to alignment-free trees inferred under TK4 and JC models using FastME 2.0.

When analyzing Yeast assemblies, NSB-TK4 and Jellyfish-JC produce a phylogenetic tree identical to the reference phylogeny (Supplementary Fig. S5). However, Skmer-JC distances produce a tree with one branch mismatch. Although the trees inferred using NSB-TK4 and Jellyfish-JC distances are topologically identical, their branch lengths differ: NSB-TK4 trees have 16% increased tree height (Supplementary Fig. S6), indicating that the JC model likely underestimates distances. In terms of additivity, Jellyfish-JC distances have an FME of 0.0034, which is 70% higher than that of NSB-TK4 (Supplementary Table S5).

When analyzing the genome skims, the tree inferred by NSB-TK4 and Jellyfish-JC is identical to the reference phylogeny regardless of the sequencing coverage (Supplementary Fig. S5). Similar to assemblies, NSB-TK4 and Jellyfish-JC recover the reference phylogeny on Saccharomyces genome skims for all levels of coverage (Supplementary Table S5). While Skmer-JC can match the reference phylogeny on the genome skim of 2× coverage, the Skmer tree has one branch mismatch in other coverage levels. On yeast genome skims, NSB-TK4 consistently achieves the lowest FM error among the three methods tested. Furthermore, even on the shallowest genome skim data (1×) tested, the NSB tree achieves a lower FM error than JC-based method on assembled data. By contrast to NSB and Jellyfish, Skmer-JC trees have higher FM errors with increasing coverage. Nevertheless, at 8× coverage, where most k-mers in the genomes are covered by at least one read, all three methods seem to approximate their level of error on the assembled data.

## 4 Discussion

We introduced a method for computing phylogenetic distances on alignment-free data based on the time-reversible, no strand-bias, four-parameter evolutionary model, TK4. Through theoretical and empirical analyses, we explored the model conditions where the more general model TK4 offers more accurate distances than the JC model, which is the simpler yet most widely used model. As expected, the improvements are most pronounced for larger distances and more substantial deviations from the JC model assumptions.

Despite overall improvements, in the simulations based on the yeast genome, we observed conditions where the TK4 model was less accurate than the JC model it contains. Deviations from the TK4 model can explain this surprising result. Even if used as the base genome for subsequent simulations, the real genomes can violate the assumptions of our algorithm in several ways. (i) Presence of non-randomly generated repeats (e.g. recent gene duplications) causes overestimating of the Jaccard index. The probability of a *k*-mer being present in both input genomes is higher when it repeats multiple times across the genome. Our calculations only correct for these repeats when they occur randomly but not by homology. (ii) Systematic variations of *ω* across the genomes, violating i.i.d. assumptions, can create loci with increased numbers of homologous and non-homologous matches after replacement. (iii) Presence of *k*-mer motifs can invalidate assumptions of Lemma 1. While some of these issues also violate JC assumptions, NSB-TK4 may be less robust to these violations than JellyFish-JC due to the more complex equations or the more complex estimation procedure (e.g. letter replacement) used by NSB.

More broadly, while the TK4 model is more complex than JC, relevant processes are also missed by TK4. An important aspect of molecular evolution we did not model is the rate heterogeneity among sites. Leading alignment-based phylogenetic estimation tools model the heterogeneity using a discrete or continuous gamma distribution. JC model can be extended to support Gamma-distributed rates (Nei and Gojobori, 1986) if the parameters of the Gamma model are known. With GTR-based simulations, we showed that TK4 is robust to model misspecification. One question is whether TK4 distances are accurate in data simulated under GTR + Γ model of evolution. Furthermore, it may be possible to incorporate a measure of rate variation in the TK4 formula (2) as well. We leave these questions to future work.

By relying on the (containment) Jaccard index similar to Mash (Ondov *et al.*, 2016) and Skmer (Sarmashghi *et al.*, 2019), NSB enables application to both assembled genomes and NGS reads in an assembly-free fashion. Interestingly, our results showed high levels of accuracy with shallow coverage (e.g. 1X) in computing distances, as demonstrated by the low FME values obtained on the yeast dataset. Thus, beyond phylogenetic inference, other applications such as species identification using genome skims can benefit from NSB.

Using *k*-mers is not the only option for distance calculations. For example, tools like pyANI (Pritchard *et al.*, 2016) and Co-phylog (Yi and Jin, 2013) estimate the distance between two genomic sequences by efficiently finding local alignments. It is possible to infer substitution probabilities from these local alignments and calculate evolutionary distance according to the TK4 model. While such approaches will not be fully alignment-free, future work should compare these methods to our proposed approach. However, even if accurate, such methods cannot be incorporated into the analyses of low-coverage short-read NGS data mentioned above when assembly is impossible.

In the scenario where assembly and alignment are available, NSB can be compared to the standard alignment-based methods for distance and phylogeny estimation. A careful comparison would require far more complex simulation pipelines—as our existing simulations do not handle indels and rearrangements. As stated earlier, alignment-free methods can improve accuracy when rearrangements make it hard to create reliable alignments; phylogenomic analyses often remove large chunks of the genome and focus on parts that are easier to align. If alignment-free methods can incorporate more complex models than currently possible, perhaps they can surpass alignment-based methods by using all the data. We believe reaching that goal will require further increases in the model complexity of alignment-free methods.

Due to the exact computation of *k*-mer counts, NSB and JellyFish can both have substantial running times. Running time for NSB scales linearly with the input genome size (Supplementary Fig. S7). On two random genomes of length 100 Mb, NSB completes within 11 min where 7 min is spent preprocessing the samples and computing the encodings and <4 min for computing all four Jaccard values and the pairwise TK4 distance. Running time for Jellyfish is about a quarter of NSB since it requires the computation of a single Jaccard value. Jaccard indices can be estimated accurately without looking at all *k*-mers using the MinHash sketching technique (Ondov *et al.*, 2016) that dramatically improves the running time, disk space and memory usage. For instance, for the fixed sketch size, Skmer completes under 15 s on the same two random genomes of length 100 Mb (Supplementary Fig. S7). However, we saw that for large distances where Jaccard is small, MinHash sketching fails. This limitation may be alleviated with newer methods such as Dashing (Baker *et al.*, 2019). Nevertheless, for smaller distances where it is accurate, we could incorporate sketching into NSB. In preliminary tests, we saw that while the main Jaccard index is often computed accurately using sketching, the replaced Jaccard indices can have consequential error levels. This reduced accuracy is likely because hash functions used in existing tools assume four letters and need to be updated for genomes with replaced letters. It may even be possible to compute all four Jaccard indices without actually replacing letters by defining hash functions that do not distinguish letters. Finally, NSB may be able to use compressed *k*-mer sets (Rahman *et al.*, 2021) to reduce its storage while keeping the same accuracy. We leave the exploration of these avenues to further work.

## Supplementary Material

vbac055_Supplementary_Data

## Data Availability

The data underlying this article are available in Zenodo, at https://doi.org/10.5281/zenodo.6974987 and https://doi.org/10.5281/zenodo.6975011.
