# Supplementary material for: Genome-wide alignment-free phylogenetic distance estimation under a no strand-bias model
Source: Bioinform Adv. 2022 Aug 12;2(1):vbac055. doi: 10.1093/bioadv/vbac055 (PMC9383262; doi:10.1093/bioadv/vbac055)
Supplement: vbac055_Supplementary_Data [file vbac055_supplementary_data.pdf]

Bioinformatics Advances, 2022, pp. 1–8

doi: See main paper
Supplementary material

SUPPLEMENTARY MATERIAL

Supplementary material for Genome-wide alignment-free phylogenetic distance estimation under a no strand-bias model

Metin Balaban,[1] Nishat Anjum Bristy,[2] Ahnaf Faisal,[2] Md. Shamsuzzoha Bayzid[2] and Siavash Mirarab[1,4,*]

[1]Bioinformatics and System Biology Program, University of California San Diego, 9500 Gilman Dr., 92093, CA, United States, [2]Computer Science and Engineering, Bangladesh University of Engineering and Technology, , 1205, Dhaka, Bangladesh and [4]Electrical and Computer Engineering, University of California San Diego, 9500 Gilman Dr., 92093, CA, United States

[*]Corresponding author. smirarab@ucsd.edu[†]These authors made equal contributions.

FOR PUBLISHER ONLY Received on Date Month Year; revised on Date Month Year; accepted on Date Month Year

Abstract

While alignment has been the dominant approach for determining homology prior to phylogenetic inference, alignment-free methods can simplify the analysis, especially when analyzing genome-wide data. Furthermore, alignment-free methods present the only option for emerging forms of data, such as genome skims, which do not permit assembly. Despite the appeal, alignment-free methods have not been competitive with alignment-based methods in terms of accuracy. One limitation of alignment-free methods is their reliance on simplified models of sequence evolution such as Jukes-Cantor. If we can estimate frequencies of base substitutions in an alignment-free setting, we can compute pairwise distances under more complex models. However, since the strand of DNA sequences is unknown for many forms of genome-wide data, which arguably present the best use case for alignment-free methods, the most complex models that one can use are the so-called no strand-bias models. We show how to calculate distances under a four-parameter no strand-bias model called TK4 without relying on alignments or assemblies. The main idea is to replace letters in the input sequences and recompute Jaccard indices between k-mer sets. However, on larger genomes, we also need to compute the number of k-mer mismatches after replacement due to random chance as opposed to homology. We show in simulation that alignment-free distances can be highly accurate when genomes evolve under the assumed models and study the accuracy on assembled and unassembled biological data. Our software is available open-source at https://github.com/nishatbristy007/NSB.

Key words: Alignment-free distance calculations, Genome skimming, TK4 model, no strand bias, Assembly-free phylogenetics

© The Author 2022. Published by Oxford University Press. All rights reserved. For permissions, please e-mail: journals.permissions@oup.com

Supplementary Methods

Sarmashghi *et al., 2019* showed that genomic distance $\hat{d}$ can be estimated on genome skim data as follows:

$$\hat{d} = 1 - \Big(\frac{(\zeta_1 L_1 + \zeta_2 L_2)\mathrm{C}}{\eta_1 \eta_2 (L_1 + L_2)}\Big)^{\frac{1}{k}} \tag{S1}$$

where ζ_i and η_i are functions of sequencing error rate, coverage, read length, and k (detailed definition of these variables are given in the original paper). We add the correction for background matches to this equation:

$$\hat{d} = 1 - \Big(\frac{(\zeta_1 L_1 + \zeta_2 L_2)(\mathrm{C} - \mathbb{E}[\tilde{\mathrm{C}}])}{\eta_1 \eta_2 (L_1 + L_2)}\Big)^{\frac{1}{k}} \tag{S2}$$

We use this equation for not just $\hat{d}$ but also $\hat{d}_{AC}$,$\hat{d}_{AG}$,$\hat{d}_{AT}$, and $\hat{d}_{CG}$ values.

Supplementary Figures

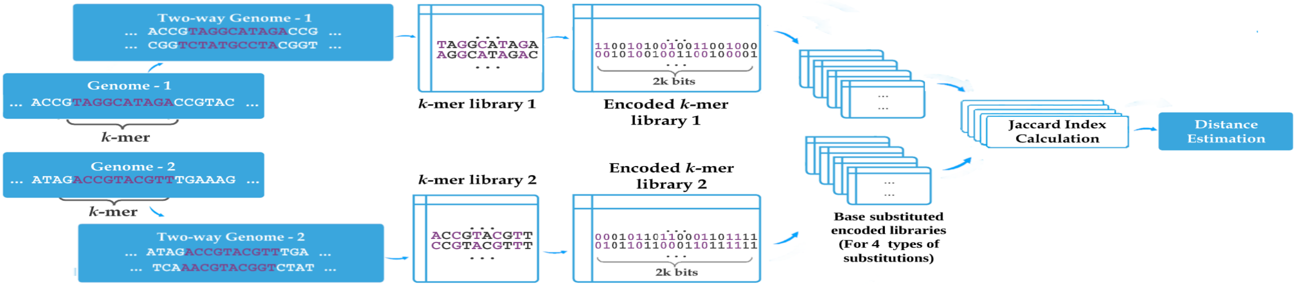

Fig. S1. Schematic diagram of our proposed pipeline. We start with adding RC of sequences followed by decomposing them into fixed-length k-mers. Next, the bases in k-mers are encoded as bitmasks and thus an encoded k-mer library is constructed. For each $(i, j) \in \{(A, C), (A, G), (A, T), (C, G)\}$, we replace the encoded bits of base i with the encoded bits of j, producing 4 base-substituted encoded libraries. Finally, using these encoded libraries, Jaccard indices and distances are estimated under the assumptions of the TK4 model and using the equations derived and presented in the main paper.

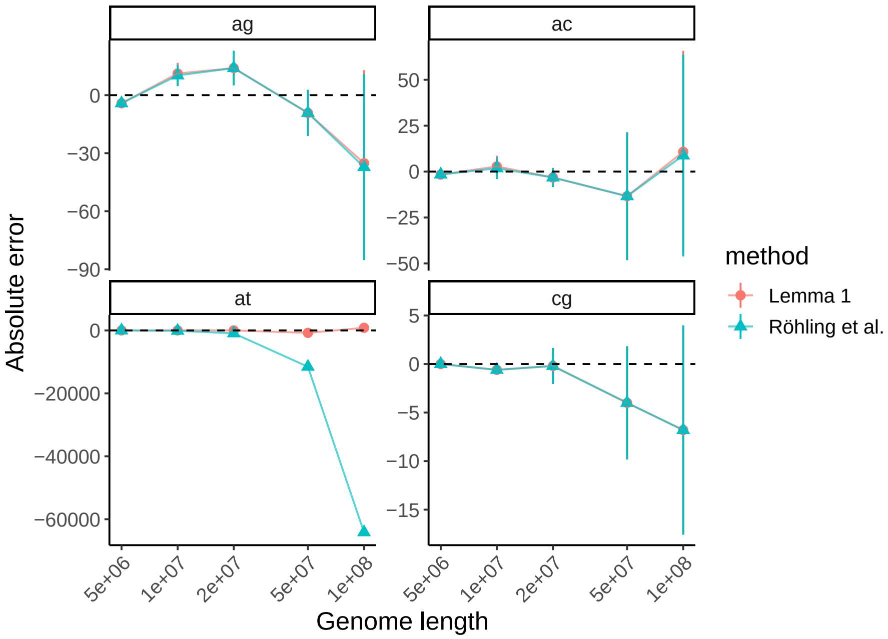

Fig. S2. Estimating number of non-homologous (by random chance) matches between two genomes after replacements. Two input genomes are created according to an i.i.d. process with base frequencies $\pi = \left[\pi_A = 0.3 \quad \pi_C = 0.2 \quad \pi_G = 0.2 \quad \pi_T = 0.3\right]$. Replacements are performed after 2-way genomes are constructed.

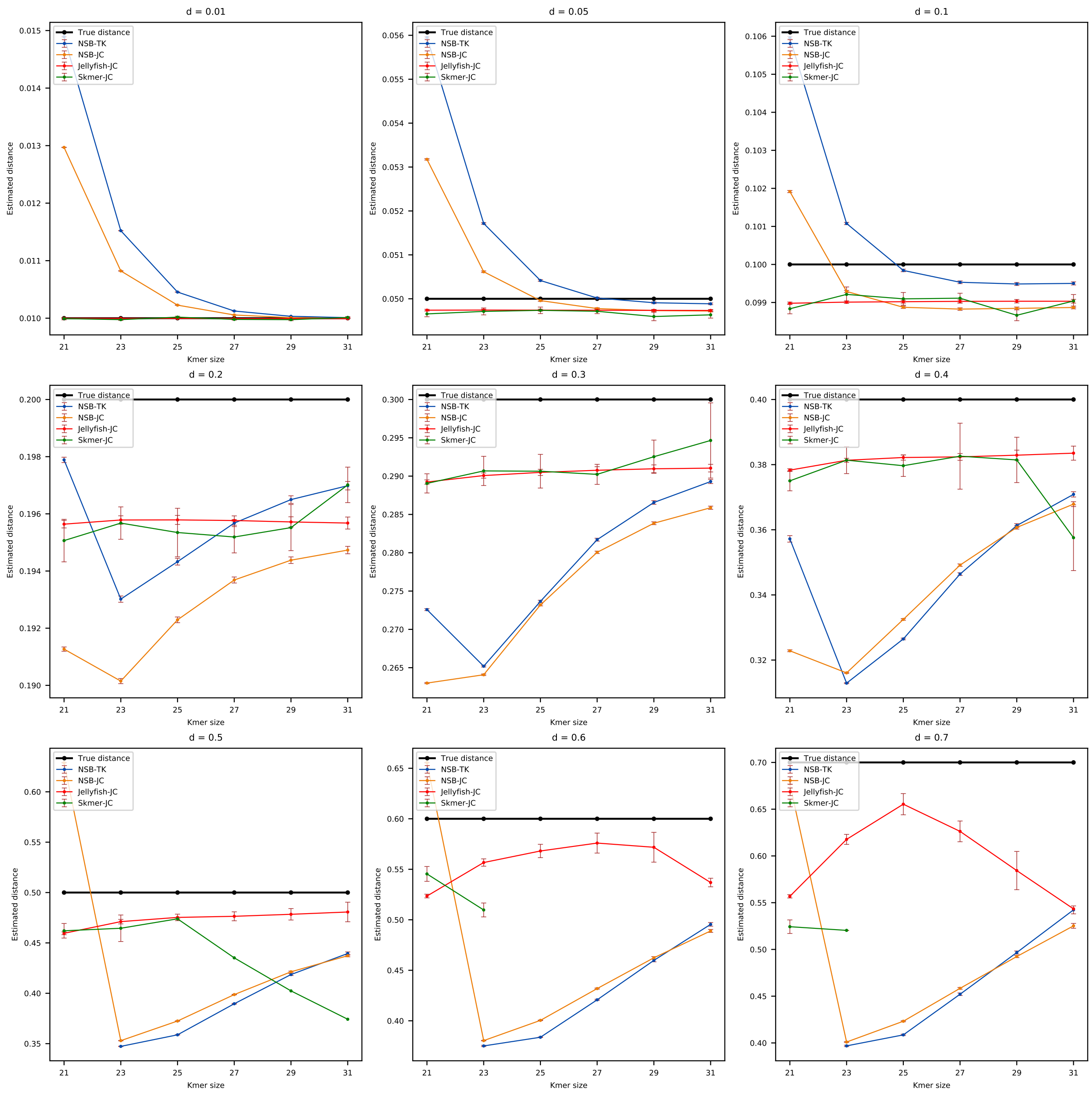

Fig. S3. The effect of parameter k on accuracy in simulated yeast genomes. Genomes are simulated using TK4 model with parameters $\alpha = 1$, $\delta = 1$, and $\gamma = 4$. Skmer does not return valid distances in $d \geq 0.6$ and $k \geq 25$ setting. NSB-TK4 does not return valid distances in $d \geq 0.5$ and $k = 21$ setting. Error bars show confidence interval of the estimate over 10 replicates.

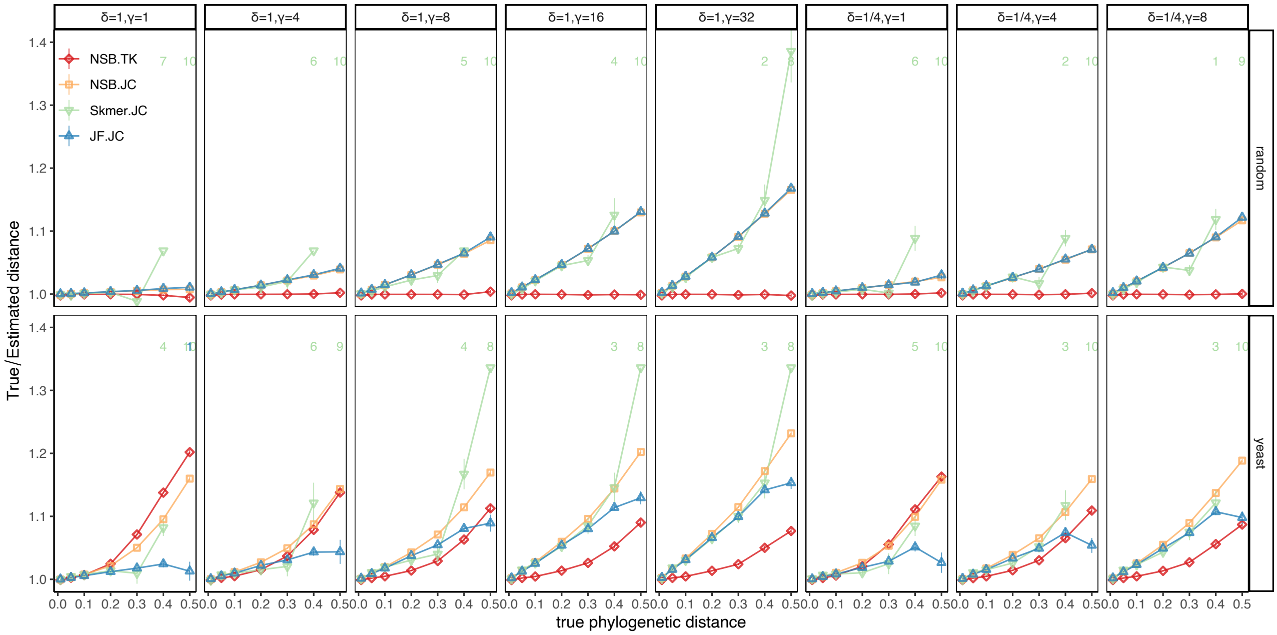

Fig. S4. Comparing the accuracy of distances estimated by different approaches on random and Yeast-based simulated genomes. Genome sequences were simulated by randomly substituting the genome skims of *Saccharomyces arboricola* (11 Mbp) and a random 100 Mbp sequence with eight different sets of TK4 parameters and with seven controlled true distances. Here, ω is fixed, and since these rates do not have a scale, $\alpha = 1$ in all cases. We show the average true distance divided by estimated distances (y-axis) with standard errors (over replicates, requiring at least two) against the true distances. Annotated numbers show the number of replicates out of 10 where Skmer or JellyFish return infinity.

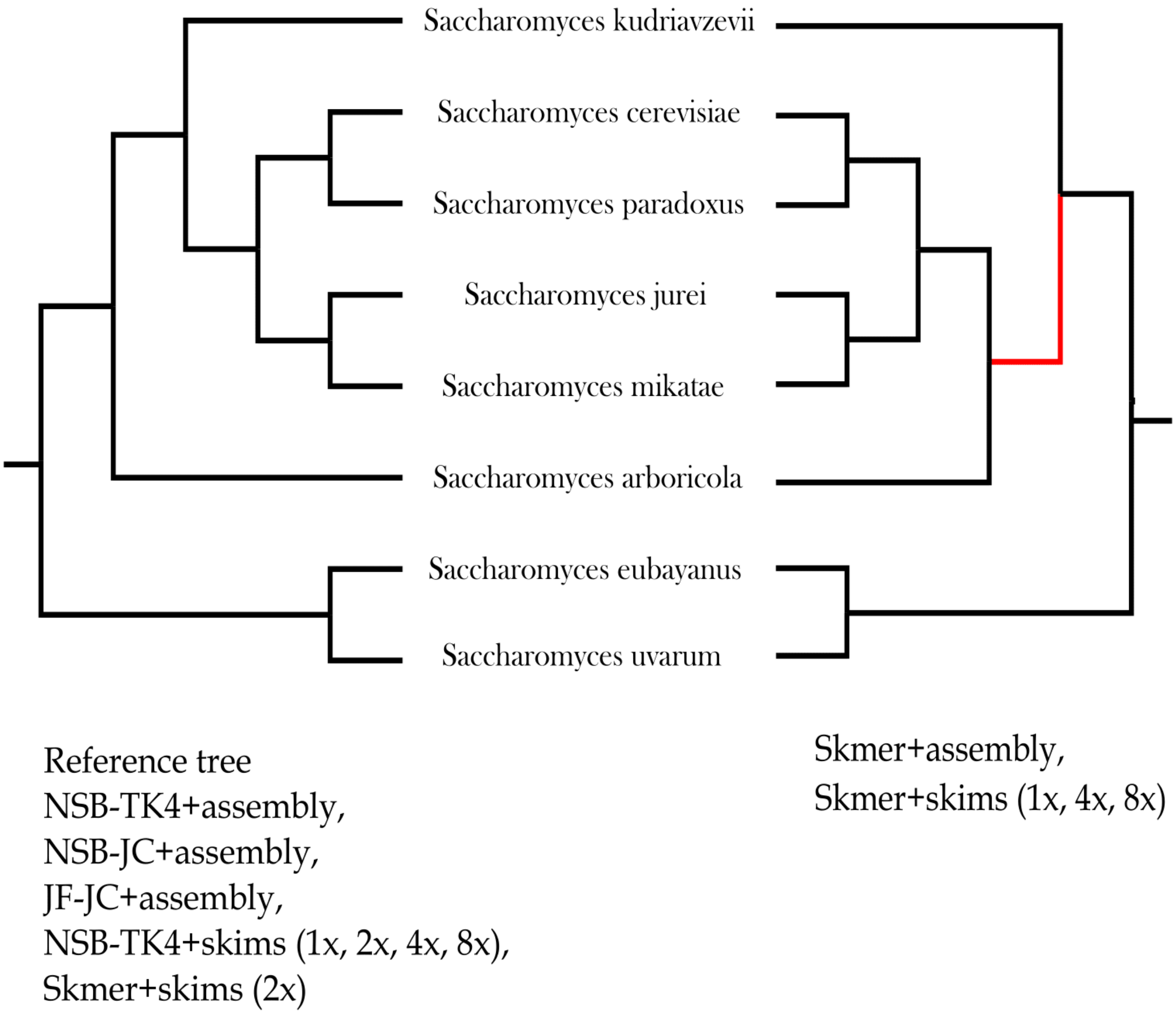

Fig. S5. Analysis of the yeast genome assemblies and skims using distances estimated under JC and TK4 models. The branch in the estimated tree which is not found in the reference tree is shown in red.

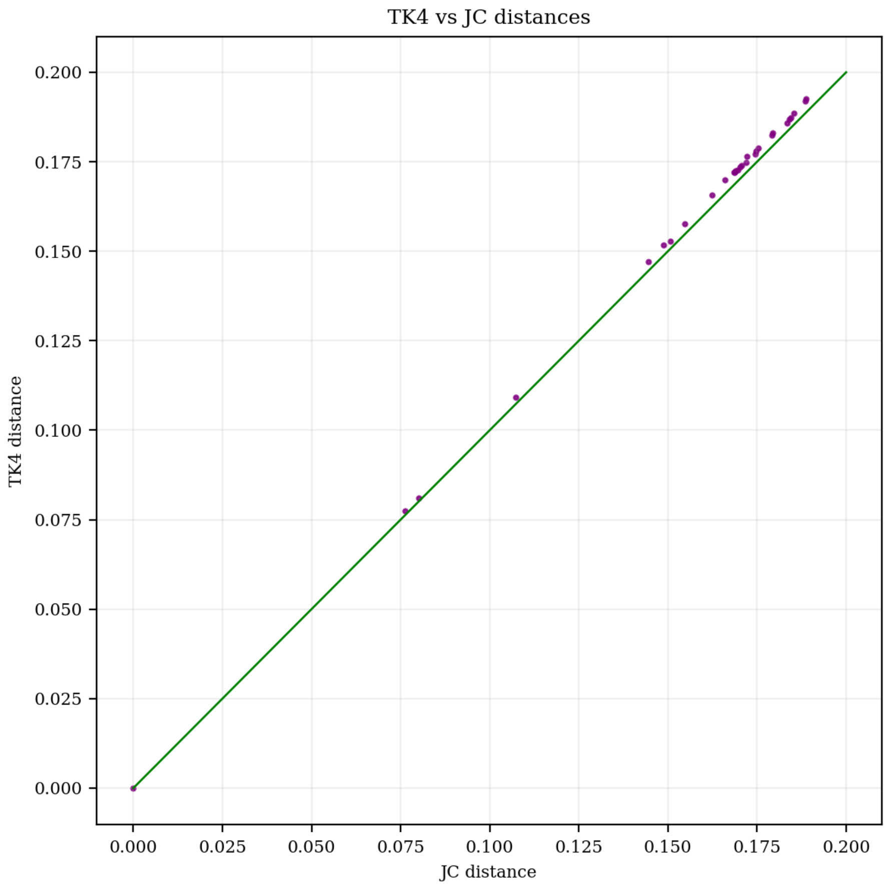

Fig. S6. Comparison of pairwise distances estimated using TK4 and JC models on 8 real Yeast genomes. TK4 distances are always slightly higher than JC.

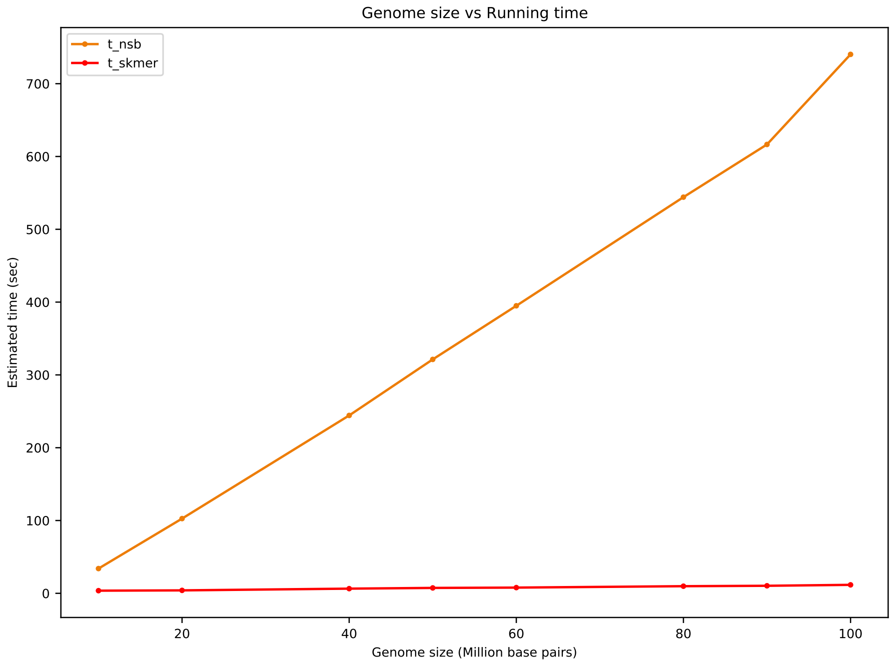

Fig. S7. Runtime comparion between NSB and Skmer in seconds. Sketch size for Skmer is fixed.

Supplementary Tables

Table S1. Different model conditions (MC), used for simulating genome sequences

.

MC	1	2	3	4	5	6	7	8	9
α	1	1	1	1	1	1	1/4	1	1
δ	1	1/4	1	1	1/4	1	1/4	1/4	1
γ	16	4	4	32	8	8	1	1	1

Table S2. Comparison of different methods to the ASTRAL tree on 10 sets of bacterial dataset. Best results are shown in bold.

	# taxa (branches)	Number of branch mismatches with ASTRAL			
		NSB-TK4	NSB-JC	Jellyfish-JC	Skmer-JC
Set 1	34(31)	**9**	11	15	13
Set 2	43(40)	11	11	**10**	**10**
Set 3	32(29)	**5**	**5**	8	8
Set 4	38(35)	**7**	**7**	8	8
Set 5	43(40)	**11**	12	12	12
Set 6	46(40)	**14**	**14**	**14**	**14**
Set 7	34(31)	**5**	**5**	6	6
Set 8	41(38)	16	16	**14**	15
Set 9	39(36)	**7**	**7**	**7**	**7**
Set 10	86(83)	**35**	37	39	36
**Sum**	433 (403)	**120**	125	133	129

Table S3. Distance error (deviation from additivity) for NSB-TK4 and Jellyfish-JC on bacterial dataset.

	# taxa (branches)	TotalFM (NSB-TK4)	totalFM (Jellyfish-JC)	TotalOLS (NSB-TK4)	TotalOLS (Jellyfish-JC)
Set 1	34(31)	**0.8209**	1.29	**0.032**	0.0436
Set 2	43(40)	**0.8032**	0.8686	**0.0114**	0.0121
Set 3	32(29)	**0.2758**	0.3534	**0.0132**	0.0139
Set 4	38(35)	**1.7227**	1.926	0.1134	**0.094**
Set 5	43(40)	**2.3761**	2.5785	0.1688	**0.1367**
Set 6	46(40)	**2.7291**	2.9287	0.2003	**0.1633**
Set 7	34(31)	**0.6379**	0.8103	**0.0309**	0.0323
Set 8	41(38)	**1.8973**	2.0946	0.1186	**0.0893**
Set 9	39(36)	**0.9773**	1.2537	**0.0478**	0.0494
Set 10	86(83)	**42.0776**	55.9276	**3.4154**	4.0831
**Sum**	433 (403)	**54.3179**	70.0314	**4.1518**	4.7177

Table S4. GenBank accession numbers of yeast species/strains.

Species/Strains	GenBank accession
Saccharomyces arboricola	GCF_000292725.1
Saccharomyces cerevisiae	GCF_000146045.2
Saccharomyces eubayanus	GCF_001298625.1
Saccharomyces jurei	GCA_900290405.1
Saccharomyces kudriavzevii	GCA_900682665.1
Saccharomyces mikatae	GCA_000167055.1
Saccharomyces paradoxus	GCA_002079145.1
Saccharomyces uvarum	GCA_002242645.1

Table S5. Topological and distance error for NSB-TK4, Jellyfish-JC, and Skmer-JC on yeast dataset.

	Coverage	RF (NSB-TK4) (Jellyfish-JC)	RF (Skmer-JC)	TotalFM (NSB-TK4)	TotalFM (Jellyfish-JC)	TotalFM (Skmer-JC)
Genome skim	1×	0	1	**0.0028**	0.0050	0.0063
Genome skim	2×	0	0	**0.0024**	0.0039	0.0072
Genome skim	4×	0	1	**0.0023**	0.0039	0.0083
Genome skim	8×	0	1	**0.0019**	0.0034	0.0084
Assembly	n.a.	0	1	**0.0020**	0.0034	0.0082
